# Immune responses at brain barriers and implications for brain development and neurological function in later life

**DOI:** 10.3389/fnint.2013.00061

**Published:** 2013-08-23

**Authors:** Helen B. Stolp, Shane A. Liddelow, Inês Sá-Pereira, Katarzyna M. Dziegielewska, Norman R. Saunders

**Affiliations:** ^1^Department of Perinatal Imaging and Health, King's College LondonLondon, UK; ^2^Department of Physiology, Anatomy and Genetics, University of OxfordOxford, UK; ^3^Department of Neurobiology, Stanford UniversityStanford, CA, USA; ^4^Department of Pharmacology and Therapeutics, University of MelbourneParkville, VIC, Australia; ^5^Department of Pharmacology, University of OxfordOxford, UK

**Keywords:** blood-brain barrier, choroid plexus, cerebrospinal fluid, inflammation, development

## Abstract

For a long time the brain has been considered an immune-privileged site due to a muted inflammatory response and the presence of protective brain barriers. It is now recognized that neuroinflammation may play an important role in almost all neurological disorders and that the brain barriers may be contributing through either normal immune signaling or disruption of their basic physiological mechanisms. The distinction between normal function and dysfunction at the barriers is difficult to dissect, partly due to a lack of understanding of normal barrier function and partly because of physiological changes that occur as part of normal development and ageing. Brain barriers consist of a number of interacting structural and physiological elements including tight junctions between adjacent barrier cells and an array of influx and efflux transporters. Despite these protective mechanisms, the capacity for immune-surveillance of the brain is maintained, and there is evidence of inflammatory signaling at the brain barriers that may be an important part of the body's response to damage or infection. This signaling system appears to change both with normal ageing, and during disease. Changes may affect diapedesis of immune cells and active molecular transfer, or cause rearrangement of the tight junctions and an increase in passive permeability across barrier interfaces. Here we review the many elements that contribute to brain barrier functions and how they respond to inflammation, particularly during development and aging. The implications of inflammation–induced barrier dysfunction for brain development and subsequent neurological function are also discussed.

## Introduction

It is a long-held belief that the central nervous system (CNS) is an immune-privileged site, due in part to the muted inflammatory response and presence of several protective brain barriers at the CNS-peripheral interface. In contrast to this earlier dogma, it is now evident that the CNS does contain immune capabilities, and that neuroinflammation is likely to play an important role in most, if not all, neurological disorders. In addition, the protective barriers of the brain contribute to these altered functions through either normal immune signaling, or disruption of the basic physiological barrier mechanisms. Recent work has shown that the peripheral immune response contributes to neuroinflammatory conditions (Anthony et al., 2011). This has been particularly well-established in conditions such as multiple sclerosis (and the corresponding animal model—experimental autoimmune encephalomyelitis, EAE) and similar findings have been reported in models of amyotrophic lateral sclerosis, stroke, and epilepsy among others (Campbell et al., 2007b, 2010; Serres et al., 2009; Auvin et al., 2010).

In all these conditions, changes in blood-brain barrier structure and function have been reported. The brain barriers play an important role in maintaining the homeostatic environment of the CNS, and damage to the various structural and functional components of the barrier systems may contribute significantly to disease etiology or progression. What is currently unclear is how (a) the brain barriers themselves contribute to inflammatory signaling in neurological disease? and (b) which specific barrier mechanisms are altered in response to inflammation? Of particular interest to us is the importance of these pathological mechanisms in the developing brain.

In a typical adult inflammatory state, cells mediating the inflammatory response arrive at the site of inflammation or infection and release a large number of mediators that act to control the accumulation and activation of other cell types (both locally and migrating). The key features of CNS inflammation include a range of responses: glial activation, edema, major histocompatibility complex expression, systemic acute phase response (general inflammation and acute phase protein synthesis), complement activation, synthesis of inflammatory mediators (e.g., cytokines, free radicals, prostaglandins) expression of adhesion molecules and the invasion of circulating immune cells (Perry et al., 1995). Due to the protective nature of both the blood-brain and blood-CSF barriers, there are two questions for consideration. Firstly, are mediators of the inflammatory response captured within the CNS space unable to be removed rapidly by the bloodstream? And secondly, does the functional tightness of the barrier impede the entry of immune cells, thereby slowing the immune response? Though the brain can mount its own defense by the activation of resident cells such as astrocytes and microglia (both cell types that are able to produce and secrete and number of cytokines), there is still a major reliance on peripheral immune cells. There is a continued argument about the balance between protection and damage in the CNS that results from a neuroinflammatory response, given its limited regenerative capacity (Aguzzi et al., 2013).

Differentiating the role of inflammatory mediators in pathogenesis is particularly difficult early in development, as a number of immune mediators play an important role in normal brain development. Neuropoietic cytokines contribute to proliferation of neural precursors, fate determination and differentiation, migration of neurons and glia, as well as cell survival and activity-dependent alteration of synaptic function (Stolp, 2013). Inflammation during development therefore, may cause widespread injury to the brain—not only due to the damaging effects of the inflammatory response itself, but also by interfering with the normal balance of cytokine signaling and therefore CNS development.

It is now well-documented that the barrier systems in the brain are well-established during early development and are essential for the normal functioning of the brain (see Saunders et al., 2012 for review). However, other research suggests that the brain barriers may be more susceptible to inflammation-induced changes in the developing brain (Anthony et al., 1997; Stolp et al., 2005a) in turn contributing to the pathology of serious neurodevelopmental disorders such as autism, cerebral palsy and epilepsy (Stolp and Dziegielewska, 2009). Alterations in signaling through barrier systems following inflammatory injury may lead to changes in many elements of brain development—contributing to these serious developmental disorders [reviewed by Stolp (2013)] or they may change the susceptibility of the brain to later onset conditions such as schizophrenia or neurodegenerative disease [reviewed by Stolp and Dziegielewska (2009); Bilbo and Schwarz (2012)].

The aim of this review is to introduce the brain barrier mechanisms and the response and contribution of these barriers to inflammation in the CNS. We shall initially discuss these issues in the context of adult disease, before exploring the developmental barrier systems and their contribution to neurodevelopmental disorders.

## Barrier mechanisms in the adult and developing brain

**Figure 1:**
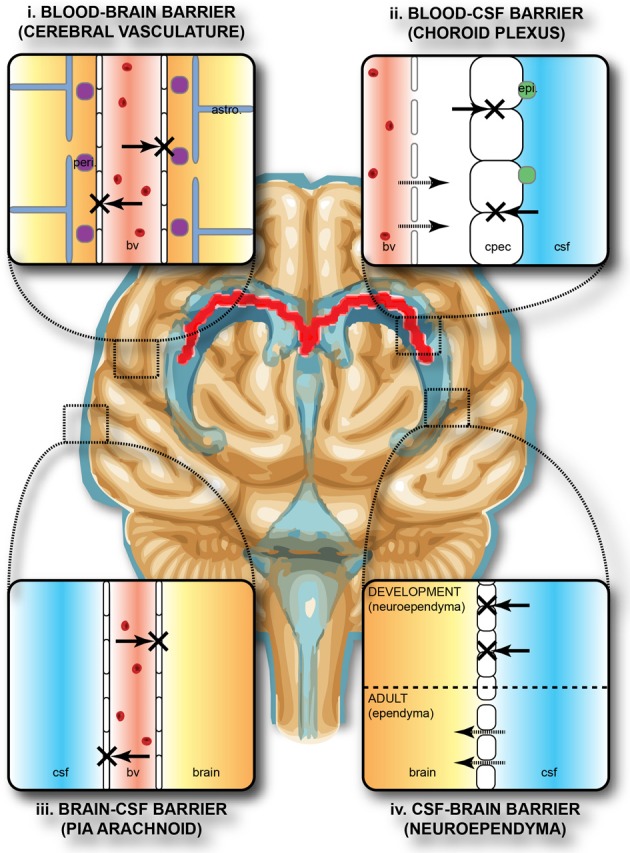
**Protective barriers of the brain.** The collective term “blood-brain barrier” is used to describe four main interfaces between the central nervous system and the periphery. (i) The blood-brain barrier proper formed by tight junctions between the endothelial cells of the cerebral vasculature. It is thought that pericytes (peri.) are sufficient to induce some barrier characteristics in endothelial cells, while astrocytes (astro.) are able to maintain the integrity of the blood-brain barrier postnatally. (ii) The blood-CSF barrier formed by tight junctions between epithelial cells of the choroid plexus epithelial cells (note the plexus vasculature is fenestrated). Resident epiplexus (epi.) immune cells are present on the CSF-surface of the plexus epithelium. (iii) The outer CSF-brain barrier and the level of the pia arachnoid, formed by tight junctions between endothelial cells of the arachnoid vessels. (iv) The inner CSF-brain barrier, present only in early development, formed by strap junctions between the neuroependymal cells lining the ventricular surfaces. In the adult this barrier is no longer present. Both the blood-brain and CSF-brain barriers extend down the spinal cord. The CSF-filled ventricular system is depicted in blue, while CNS brain tissue is in brown. The lateral ventricular choroid plexuses are shown in red. Abbreviations: astro, astrocyte; bv, blood vessel; cpec, choroid plexus epithelial cell; csf, cerebrospinal fluid; peri, pericytes.

The brain develops and functions within a well-defined internal environment, which is determined by regulation of interchange between the main compartments of the CNS, brain, cerebrospinal fluid (CSF) and the blood, by a combination of physical and functional mechanisms. These mechanisms, often referred to by the generic term of “blood-brain barrier,” are present at three main interfaces in the brain, both in the adult and in the embryo, although there are some important age-related differences between them. These interfaces, illustrated in Figure 1, are: (i) the blood-brain barrier proper at the level of the cerebral endothelial cells, (ii) the blood-CSF barrier at the epithelial cells of the choroid plexuses within the four cerebral ventricles and (iii) the pia arachnoid. There is also an additional barrier interface (iv), present only in the early brain development, between the CSF and the brain interstitial fluid. In both the adult and developing brain the essential morphological feature of the blood-brain barrier proper (i) lies in the presence of tight junctions between the cerebral endothelial cells of the vasculature of the brain both within the parenchyma and over the surface in the pia-arachnoid. Compared with other blood vessels there is also a lack of pinocytotic vesicles in the cerebral endothelial cells, although there is some evidence that they may be more frequent in endothelial cells in the developing brain (Dziegielewska et al., 1979). In the choroid plexuses (ii), tight junctions are found between intimately apposed epithelial cells. The tight junctions prevent the intercellular (paracellular) passage of small molecules even in the very early stages of the developing brain (Ek et al., 2003, 2006). An important functional consequence of this is that the presence of tight intercellular junctions enables the cerebral endothelial cells and choroid plexus epithelial cells to have effective one-way transport mechanisms (Liddelow et al., 2009; Ek et al., 2010), which are essential for establishing and maintaining the internal environment of the brain separate from that of the rest of the organism. The morphology of the barrier interface over the surface of the brain (iii) is more complex during early development. Thus, in addition to the adult barrier of tight junctions linking the endothelial cells of blood vessels in the pia arachnoid, there is a wide array of specialized intercellular junctions over the pial surface of the brain, which has been described in the rat embryo (Balslev et al., 1997). From embryonic day 14 (E14), the progressive appearance of distinct junctional structures between the glial end feet was observed. Analysis of albumin distribution at the electron microscopic level suggested that these junctions may contribute to restriction of diffusion between the subarachnoid space and the brain interstitial space. However, at E12 and E14, the intercellular basis for this barrier appeared incomplete, so it was suggested that basement membrane may be an important component of this functionally effective barrier interface (Balslev et al., 1997). At the CSF-brain interface (iv) lining the cerebral ventricles, early in embryonic development, the cells of the neuroependyma (neuroepithelium) are linked by strap junctions (Møllgård et al., 1987), which are an effective limitation to intercellular diffusion at least for large molecules (Fossan et al., 1985). During brain development these strap junctions disappear, and in the adult the cells lining the ventricles (ependymal cells) are linked by gap junctions (Møllgård et al., 1987) that do not provide a significant restraint to diffusion of even large molecules from CSF to brain interstitial fluid (Fossan et al., 1985). Consequently, in the embryonic brain only, there appear to be barrier mechanisms that restrict entry of proteins from CSF into the brain interstitial fluid. These proteins may be contributing to some aspects of early brain development such as neurogenesis and cellular differentiation in the ventricular zone (VZ), either by uptake of individual proteins or ligands bound to them (see below).

In terms of functional exchanges at the brain barrier interfaces, these have only been studied in any detail at the blood-brain and blood-CSF barriers, although much less so during early development than in the adult brain. Some recent studies have used molecular screening techniques to elucidate the range of genes coding for various proteins involved in transport mechanisms that are expressed at these interfaces during fetal or neonatal stages of brain development. Daneman et al. (2010) used Affymetrix genechip arrays to compare expression patterns in neonatal and adult mouse cerebral endothelial cells, while Liddelow et al. (2012, 2013) used both Affymetrix arrays and high-throughput RNA sequencing to compare gene expression in mice and rats from E15 embryos and adult choroid plexuses. These studies are complimented by those of Kratzer et al. (2012) who used Affymetrix arrays to study gene expression in rat choroid plexus at several ages between E18 and adult, and by Marques et al. (2009) of adult mouse choroid plexus using Illumina whole genome beadchips. Collectively, these studies have revealed expression of an astonishing array of transcripts for proteins known to be associated with tight junctions, transporters (both influx and efflux) and ion channels, as well as numerous enzymes in various metabolic and signal transduction pathways. Many of these genes are expressed at a higher level (in some cases two orders of magnitude higher) in the developing cerebral endothelial and choroid plexus epithelial cells than in the adult. A major problem, however, is determining whether these high levels of expression also reflect a higher level of transport. By comparing published data from *in vivo* transport from blood to brain or CSF, for example for glucose and amino acids in neonatal animals (such experiments in fetuses have so far proved to be a technically intractable problem), it is clear that these high levels of expression are likely to reflect higher levels of transport in the developing brain (Saunders et al., 2013). However, because of the overlap in substrates for different transporters it is not yet possible to be sure that higher expression always equates to greater transport. Some examples comparing expression of individual transporter genes with data on *in vivo* transport of various amino acids are shown in Table 1.

**Table 1:** **Comparison of expression of influx transporters and published reports on transport function in the developing brain**.

**Transporter**	**Fold change**	**Transport function**
*Slc16a10*	66.8	Iodothyronines T3, T4^a^
*Slc6a15*	11.4	Neutral amino acids^b^
^*^Slc40a1	9.6	Iron^c^
*Slc7a11*	7.1	Cysteine, glutamate^b^
*Slc4a1*	5.5	Anion transporter^d^, (Cl^−^-HCO_3_ exchange)^e^
*Slc6a13*	4.6	GABA transporter^f^
*Slc1a4*	4.4	Glutamate, neutral amino acids^g^
*Slc38a4*	4.2	Acidic and neutral amino acids^b^,^g^
*Slc6a6*	4.1	Taurine^b^
*Slc4a4*	4.1	Na^+^-HCO^−^_3_ cotransporter^d^
*Slc7a1*	4.1	Acidic amino acids^b^
*Slc39a8*	3.3	Zinc transporter^h^

Expression levels for the E15 and adult mouse choroid plexus. Fold change in expression compares E15 with adult choroid plexus (positive values are enriched in the embryo). Superscript numbers indicate published studies showing transport into developing brain or CSF.

* Gene product ferroportin-1 identified in choroid plexus. References:

a (Porterfield and Hendrich, 1992);

b (Lefauconnier and Trouve, 1983);

c (Morgan and Moos, 2002);

d (Damkier et al., 2010);

e (Amtorp and Sorensen, 1974);

f (Al-Sarraf et al., 1997);

g (Al-Sarraf, 2002);

h (Chowanadisai et al., 2005). Data from Liddelow et al. (2012), adapted from Saunders et al. (2013).

Early in brain development, the choroid plexuses are much more substantial structures compared to the limited level of vascularization of the brain (Saunders et al., 2013). It seems reasonable to propose that the plexuses may be more important than the sparse blood vessels for the supply of nutrients and other essential molecules to the early developing brain [as originally proposed by Klosovskii and Zhukova (1963)]. If this is the case, it is not clear whether the access for these materials or immune cells to the CNS is via diffusion across the CSF-brain interfaces (internal and external) or if there are also transport mechanisms in the cells of these interfaces. Such a mechanism for plasma proteins has recently been proposed for choroid plexus epithelial cells Liddelow et al., (2012).

**Figure 2:**
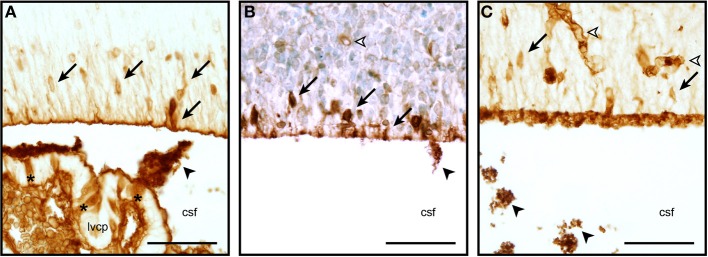
**Neuroependymal cell uptake of plasma proteins from the CSF.** Coronal, paraffin-embedded section of lateral ventricular wall from sheep fetuses at embryonic day 40 (E40, **A**), E60 **(B)**, and E15 mouse **(C)** stained to detect endogenous plasma protein. The migrating neurons in the ventricular zone are strongly stained (arrows), blood vessels also show a positive staining reaction (unfilled arrowhead). Protein is also seen precipitated in the CSF (filled arrowheads). Extensive precipitation of CSF plasma protein can be seen in the embryonic mouse example displayed in panel **(C)** (arrowheads, also in **A**,**B)**. Choroid plexus epithelial cells individually positive for protein can also be seen in panel **(A)** (asterisks). Abbreviations: csf, cerebrospinal fluid; lvcp, lateral ventricular choroid plexus. Scale bars 50 μm in each.

So very little is known about the cellular and molecular properties of the CSF-brain interface in the developing brain that is not even clear if the transport mechanisms at this interface function similarly to other barriers. It is known that at some early stages of brain development, at a time when strap junctions are present at the CSF-brain interface (see above), plasma-derived proteins found in the CSF (which are present in a much higher concentration than in adult CSF, Dziegielewska et al., 1988) are taken up by some of the neuroependymal cells lining the ventricular system (Figure 2). This phenomenon has been described for both endogenous proteins (e.g., alpha 2-HS glycoprotein in human fetuses—Møllgård et al., 1988) and exogenously administered non-native proteins (e.g., human albumin injected into the wallaby—Dziegielewska et al., 1988; or rat—Balslev et al., 1997). Examples of plasma protein staining in different animal species are shown in Figure 2. It is not known, however, if this is selective with respect to individual proteins (as is seen in the choroid plexus, Liddelow et al., 2009), regions of the ventricular system or stage of brain development. It is also unclear whether the functional significance of this protein uptake lies in the ligands known to be bound to many of these proteins (e.g., growth factors, vitamins) or in some specific properties of the individual proteins themselves. It is also unknown if this internal barrier is able to impede any CNS immune response.

As a result of the complex array of barrier mechanisms (both physical and biochemical) that surround the brain both in the adult and during development, there is great control of both the passive barrier to diffusion, and of the dynamic transport system controlling the internal environment of the CNS. As more evidence is provided for the movement of ions, plasma proteins, drugs and other molecules both into and out of the CNS, there is increasing support for the notion that an interaction with the immune system is additionally one of the many functions of these barrier systems.

## Adult response to inflammation

### Cellular infiltration into the brain

The CNS is continuously monitored by resident microglia and blood-borne immune cells such as macrophages, dendritic cells and T cells that are able to detect damaging agents that would disrupt homeostasis and optimal functioning of neurons and glia. Normal immune mechanisms in the CNS are often thought of in a manner different from that seen in the periphery—for instance the immune response in the brain can be substantial (e.g., in response to meningitis) but by contrast, a loss of immunity is also reported (e.g., cerebral infections) (for review see Ousman and Kubes, 2012) and the muted inflammatory response in the brain following injury (Andersson et al., 1992) was the original rational behind the concept of the CNS being an immune-privileged site. The developing, and ageing, brain appears to have an exacerbated immune response compared to that normally seen in the adult (Perry et al., 1995; Campbell et al., 2007a). An important example of the interaction of peripheral immune cells with the CNS is the myeloid origin of the innate CNS immune cells—microglia (Aguzzi et al., 2013). Additionally, resident bone marrow-derived perivascular cells inhabit the perivascular space, which directly communicates with the CSF-filled subarachnoid space. These cells are able to respond rapidly to inflammatory injury and are continuously replaced by peripheral monocytes (Hickey and Kimura, 1988). It is therefore essential that the brain-barriers are able to facilitate entry of these cells into the CNS as part of normal function. This mechanism for facilitating peripheral immune cell entry to the CNS, however, appears to be up-regulated in cases of neuroinflammation (e.g., during stroke and multiple sclerosis), and is associated with deleterious effects on pathology (Vexler et al., 2006; De Vries et al., 2012). There is, therefore, a fine line between a “helpful” inflammatory response to injury in the CNS and pathological neuroinflammatory state.

There is still controversy in the field whether or not resident perivascular cells are able to remove themselves from the CNS to instigate a peripheral immune reaction following failure of the CNS to mount an adequate immune response (Matyszak and Perry, 1998). It has been demonstrated that CNS-derived antigens can leave the CNS via drainage through lymph nodes—potentially making CNS antigens available to the periphery without the need for the movement of cells across the blood-brain barrier (Harling-Berg et al., 1989). An acute phase response can be initiated in the liver to induced cerebral inflammation, as a direct result of this type of signaling (Anthony et al., 2011). From recent evidence it seems more likely that perivascular cells contribute to multiple sclerosis disease progression (or EAE in animal models) by reactivation of encephalitogenic T-cells (Mcmahon et al., 2005). Multiple sclerosis is characterized by substantial diapedesis of T-cells across the blood-brain barrier, contributing to demyelinating plaques associated with the disease (Engelhardt and Coisne, 2011; Zaguia et al., 2013). It is currently unclear whether this is a normal immune/blood-brain barrier response to myelin antigen presentation (even if the presence of these antigens themselves is abnormal), or whether during diseases like multiple sclerosis they are exacerbated as a result of an abnormal and exaggerated response from the cerebral vasculature. Activated T-cells leave lymph nodes and are likely able to gain additional entry to the CNS across the choroid plexuses into CSF where they are re-stimulated by meningeal and choroid plexus antigen presenting cells (Kolmer's epiplexus macrophage cells) and produce cytokines. The mechanism for the entry of these cells across the choroid plexus is unclear, evidence supporting it has been recently reviewed by Engelhardt and Sorokin (2009). Choroid plexus epithelial cells constitutively express ICAM-1 and VCAM-2, and MADCAM1 (mucosal vascular addressin cell adhesion molecule 1) during inflammation on their apical surfaces. This localization means therefore, that they are not available for the basolateral to apical migration of the immune cells (blood-to-CSF direction; Wolburg et al., 1999). This is counterintuitive, considering the high number of leukocytes frequently observed in the CSF under neuroinflammatory conditions. A recent study has suggested CCR6^+^ T-cells may use chemokine CC ligand 20 (CCL20) expressed by the choroid plexus epithelial cells to migrate across the CNS (Reboldi et al., 2009) rather than more traditional adhesion molecules, though P-selectin has also been identified at the choroid plexus epithelial barrier (Wolburg et al., 1999). Following entry of activated T-cells into the CNS, subpial vessels are activated and expression of adhesion molecules and chemokines increases, facilitating the process of T cell entry into the CNS. Typically in a disease such as multiple sclerosis, these cells will remain in the abluminal perivascular space unless further activated by interactions with perivascular macrophages and microglia, allowing them to invade the parenchyma (Ransohoff and Engelhardt, 2012). An additional new finding proposes that the choroid plexus epithelial tight junctions lack *Claudin3* (Liddelow et al., 2013), which when knocked out in mice blood-brain barrier endothelial cells causes increased peripheral immune cell diapedesis (Wolburg et al., 2003) suggesting that the choroid plexus has the junctional make-up to allow infiltration of peripherally derived immune cells and monitoring, without a destruction of other barrier mechanisms—important for the maintenance of the CNS internal milieu.

Immune surveillance, in the absence of specific inflammatory signals is therefore likely to occur primarily through the blood-CSF barrier, facilitated by the specific composition of the junctions between epithelial cells. Importantly, junctional rearrangement appears to be an essential element of inflammation-induced cellular recruitment to the brain.

The passage of immune cells through the blood-brain barrier is not a simple process and requires a sequential interaction between different type of molecules on the surfaces of immune cells and endothelial cells. This process has been well described and extensively reviewed (see Banks and Erickson, 2010; Engelhardt and Coisne, 2011; Greenwood et al., 2011). Briefly, adhesion molecules including VCAM-1 on endothelial cells bind to leukocyte integrins, initiating a number of signaling processes that ultimately combine with a number of adhesion molecules allowing a firm attachment between the cell types, and reorganization of the endothelial cytoskeleton to allow diapedisis to occur. This may include changing the interactions between the paracellular tight junctions (Greenwood et al., 2003; Carman and Springer, 2004) facilitating diapedisis via a paracellular route, as well as transcellular route, and potentially causing increased permeability to the solutes in plasma as well as the white blood cells. To facilitate this process, inflammatory stimuli can induce redistribution of junctional adhesion molecule A (JAM-A/*F11r*) away from the cell-to-cell junctions between endothelial cells. Inhibition of this redistribution can reduce the migration of monocytes and neutrophils (Stamatovic et al., 2012), supporting the concept that rearrangement of tight junction proteins facilitates infiltration of leukocytes into the brain via a paracellular route during inflammation. The earlier onset of EAE in *Pecam1* knock-out mice also suggests that weak junctional attachments between cells may sensitize the barrier to injury (Graesser et al., 2002). However, in this instance only increased white blood cell infiltration has been reported rather than increased permeability to solutes (discussed below). Peripheral cytokines stimulate this whole process by causing endothelial cells to increase expression of cell adhesion molecules on their surface, including selectins, ICAM-1, and VCAM-1 (Meager, 1999), in turn causing alterations in the effectiveness of immune cell penetration to the CNS. It has been shown that during the induction of EAE, endothelial cells forming the blood-brain barrier display an increase in the level of CCL2 proteins (also known as monocyte chemoattractant protein 1, MCP-1). CCL2 is the ligand of the activated mononuclear immune cell receptor, CCR2, effectively increasing the number/rate entry of these peripheral immune cells across the blood-brain barrier (Sagar et al., 2012).

Upon entry to the CNS, immune cells can either set up residence in the glia limitans and monitor the health of CNS cells (as is the case with perivascular cells—see above) or they can immediately move further into the nervous tissue to stage further immune responses. If no inflammatory mediator/antigen is present upon entry to the glia limitans, these cells return to the periphery (Hickey et al., 1991). An equivalent phenomenon has been recognized in systemic vascular beds (Proebstl et al., 2012; Alon and Nourshargh, 2013).

### Changes in barrier permeability

Besides the active role of the brain barriers in immune cell infiltration into the brain, it is thought that inflammation causes pathological changes, which result in increased passive permeability of brain barrier to solutes, contributing to exacerbation of the neuro-inflammatory response. Even in multiple sclerosis, where a focus is normally placed on cellular infiltration, the disruption of the blood-brain barrier tight junctions precedes the formation of sclerotic lesions (Minagar and Alexander, 2003; De Vries et al., 2012). Altered zonular occludin 1 (ZO-1) presence in cerebral microvessels in multiple sclerosis affected brains has been observed in both normal-appearing white matter and inactive lesions, in addition to areas with active lesions (Plumb et al., 2002; Kirk et al., 2003). Altered ZO-1 presence was associated with fibrinogen entry into the brain parenchyma even in the absence of leukocyte recruitment in active lesions (Kirk et al., 2003). Altered vessel distribution of tight junction proteins has also been observed in cases of cerebral amyloid angiopathy (Carrano et al., 2012). No white blood cell infiltration has been reported associated with vessels lacking tight junction proteins, though clumps of microglia in the nearby tissue support the inflammatory nature of these changes. Increased fibrinogen in the brain parenchyma suggests, as described above for multiple sclerosis that the loss or altered distribution of tight junction proteins can be associated with increased permeability to solutes from the blood, without associated cellular infiltration (Carrano et al., 2012). The blood-CSF barrier may also be affected by inflammation in this manner, though this barrier has been less extensively studied (in terms of both solute permeability studies and assessment of tight junction integrity) compared to the blood-brain barrier. In *in vitro* experiments, choroid plexus epithelial cell monolayers showed altered tight junction protein distribution for CLAUDIN-2 and OCCLUDIN when the cells were exposed to retrovirally-activated T-cells (Khuth et al., 2005). This cellular interaction or direct administration of pro-inflammatory cytokines also caused deficits in both active influx transport and efflux systems in the choroid plexus epithelial cells. *In vivo* peripheral inflammation caused by administration of lipopolysaccharide reduced expression of *Claudin3, 5* and *11* in the adult mouse choroid plexus epithelium (Marques et al., 2009). These *in vivo* results are very useful, though it should be noted that other authors (e.g., Liddelow et al., 2013) when using next generation RNA sequencing reported no expression of *Claudin3* or *5* in the choroid plexus epithelial cells.

The mechanism by which inflammation causes this change in tight junction integrity, particularly in the absence of leukocyte infiltration, is not completely understood. A study from Rigor et al. (2012) demonstrated protein kinase C-θ (PKC-θ ) mediated barrier dysfunction via IL-1β treatment. The authors report increased PKC-θ activation in an *in vitro* model of the blood-brain barrier, followed by decreased transendothelial electrical resistance. It was proposed that ZO-1 protein phosphorylation and consequent tight junction disorganization might explain the low transendothelial electrical resistance after IL-1β exposure (Rigor et al., 2012). Although suggestive, these *in vitro* results require *in vivo* substantiation of this mechanism. PKC isozymes are capable of phosphorylating a variety of proteins that regulate diverse cellular signaling pathways: some proteins related with cytoskeleton rearrangement, which can affect tight junctions organization, may be activated as shown via TNFα modulated of p115RhoGEF phosphorylation (via PKC-α ) and consequently RhoA activation. This activation promotes F-actin rearrangement and increased endothelial cell permeability *in vitro* (Peng et al., 2011), but has not been demonstrated *in vivo*.

In addition to increased permeability to solutes through the paracellular pathway following tight junction disruption, new evidence suggests that up-regulation of pinocytotic activity may contribute to increased solutes of permeability via a trancellular route, with no alteration in tight junction morphology (Armulik et al., 2010). This phenomenon is a well-recognized transport mechanism in the other biological systems and appears to be regulated by inflammation [as described, for example by Chidlow and Sessa (2010)], and needs to be investigated further at the brain barriers.

### Inflammation induced changes in transporters at the blood-brain barriers

The endothelial cells of the blood-brain barrier and epithelial cells of the choroid plexus blood-CSF barrier are studded with many influx and efflux transporters (for review see Saunders et al., 2013) Transport across the blood–brain barrier and blood–CSF barrier is directional, with different classes of transporters involved in movement into (e.g., most SLC transporters) and out of (e.g., ABC efflux pumps) the brain. Together, these combined transporters have different effects, ranging from removing solutes from the brain, preventing their entry into the brain (efflux mechanisms), setting up ion gradients or delivering specific nutrients, ions and other required molecules to the brain cells (influx mechanisms). These transporters function normally to maintain the internal homeostatic milieu of the CNS, however, many transporters at the barrier are altered during inflammation and can contribute to the overall inflammatory response (Khuth et al., 2005; Von Wedel-Parlow et al., 2009; Erickson et al., 2012).

Alteration in the expression of barrier transporters is not consistent across diseases or disease models. In multiple sclerosis, endothelial cells display reduced expression of the efflux transporter P-glycoprotein (PGP; Kooij et al., 2010), while in the SOD1 mouse model of amyotrophic lateral sclerosis, PGP is enriched on vessels within neurodegenerative regions (Jablonski et al., 2012). In isolated rat brain capillaries, increases of both PGP activity and protein levels were observed 6 h after TNFα and endothelin-1 exposure (Bauer et al., 2007), while *in vivo* experiments in adult rats evidence suggests that inflammation after endothelin-1 intrathecal injection increases PGP and BCRP activity only, without changing the protein levels (Harati et al., 2012). In contrast, Parkinson's disease patients present with alterations in one of the active transport processes at the blood-brain barrier that associate with disease etiology. Polymorphisms in the *Abcb1* gene have been recognized in Parkinson's disease patients (Westerlund et al., 2009), and PET studies have shown decreased function of PGP in Parkinson's disease (Kortekaas et al., 2005; Bartels et al., 2008). It is hypothesized that this may contribute to pathology by increasing the cerebral burden of iron, as well as sensitizing the brain to damage following pesticide exposure (Bartels, 2011). Developmentally, there are changes in both gene expression and protein presence of many of these transporters at both the blood-CSF and blood-brain barriers. Ek and colleagues (2010) showed in the rat that PGP (*Abcb1*) expression in both the forebrain and brainstem increased between E13 embryos and adults. MRP1 (*Abcc1*) expression peaked at birth and MRP4 (*Abcc4*) at 1 week postnatally, while BCRP (*Abcg2*) levels remained constant through development. This was in contrast to the choroid plexus epithelium, which displayed a large decrease (20-fold) in *Abcg2* (BCRP) expression, and increases in *Abcc1* (MRP1) and *Abcc4* (MRP4) levels. The authors were able to also show these changes using immunohistochemistry of protein levels, suggesting the transcript was active. More recent studies show that the absolute levels of these transcripts are much higher at the choroid plexus when compared with the blood vessel endothelium (Daneman et al., 2010; Liddelow et al., 2012, 2013).

No study to date has investigated the alterations in transcript or protein following inflammation in developing brain barriers. Increased efflux activity following inflammation, for example by up-regulation of ABC transporters may be an important protective mechanism. However, increased expression does not necessarily equate to increased function. Thus, these data highlight the importance of not only looking at gene expression and protein levels, but also transporter activity to try to unveil the contribution of efflux pumps to inflammation pathophysiology. Therefore, it is important to keep in mind the different steps in the inflammatory response and the different inflammation scenarios that can modulate how brain barriers may contribute in protective or harmful ways. Thus, due to the effects of inflammation on various transporters, brain barriers can contribute to changes in the CNS environment independently, beyond the changes produced as an associated effect of the activation of immune cells within the brain.

### Inflammatory signaling in cerebral endothelial and epithelial cells

In ischemic stroke, compromised endothelial cells produce inflammatory cytokines and chemokines including the interleukins IL-1β, IL-8, and CCL2. These cytokines induce expression of cell adhesion molecules by endothelial cells, facilitating the movement of peripheral immune cells into the CNS (Stanimirovic and Satoh, 2000) and potentially contributing to the initiation of cellular responses to inflammation by microglia and astrocytes within the brain parenchyma.

Daneman et al. (2010) investigated the transcriptome of purified blood-brain barrier endothelial cells in postnatal mice and reported up-regulation of the LPS/IL-1 mediated inhibition of retinoic x receptor (RXR) pathway in brain endothelial cells when compared to lung and liver endothelium. Downstream genes of RXRα were also enriched in cerebral endothelium, while inhibitory molecules of the receptor were reported only in peripheral blood vessels. RXRα nuclear receptor can activate the transcription of numerous molecules and can be inhibited by a kinase cascade initiated by LPS, IL-1, or TNFα signals. These data suggest that the RXR pathway may play an important role in the maintenance of blood-brain barrier immunogenic properties. As outlined above, however, this pathway has not been investigated in CNS inflammation to confirm its role in blood-brain barrier function and dysfunction.

The choroid plexus blood-CSF barrier may also respond to inflammation by producing different inflammatory mediators, activating different pathways. Microarray analysis was able to identify a high number of genes that were up-regulated after peripheral injection of LPS in adult mice (Marques et al., 2009). Chemokines, including *Ccl4, Ccl5, Ccl7*, and *Cxcl1* as well as interleukins, *Il1β*, *Il6,* and *Il15*, cell adhesion molecules and many transporter molecules were enriched. Moreover, genes of the MAPK, NFκ B, interferon signaling, and IL-10 pathways were also identified.

There is increasing evidence that the brain barriers are able to mount, at least an initial response to peripheral inflammation—either in reaction to infiltration of inflammatory mediators to the CNS, or due to the effects of infiltration of activated peripheral immune cells, and that this vascular inflammatory response may in itself contribute significantly to neuroinflammatory disease.

## Developmental inflammatory mediators at the brain barriers

Recent reports on the transcriptome of the blood-brain (Daneman et al., 2010) and blood-CSF (Liddelow et al., (2012, 2013; Kratzer et al., 2013) barriers during early development highlight the alterations in expression levels of a range of transcripts that are involved in the inflammatory response. Combined with studies looking at brain barrier cells following inflammatory insults (Marques et al., 2009) it is apparent that the brain barriers are able to take a more active role in responding to both peripheral and central immune responses than previously believed. Perinatal brain injury frequently complicates preterm birth and can lead to significant long-term morbidity. Cytokines and inflammatory cells are mediators in the common pathways associated with perinatal brain injury induced by a variety of insults, such as hypoxic-ischemic injury, reperfusion injury, toxin-mediated injury, and infection—all of which cause a rapid and sometimes sustained immune response. In addition to movement of peripherally produced inflammatory mediators across the brain barriers, the infiltration of peripheral immune cells can also alter throughout development. The differential expression of *Icam1* (intercellular adhesion molecule 1) is higher at the blood-brain barrier than at the blood-CSF barrier (Daneman et al., 2010; Liddelow et al., 2012; Saunders et al., 2013). The developmental changes in expression of *Icam1* are also different between the two main brain barriers—with no developmental change in expression in the cerebral vasculature, but a slight increase in expression in the adult choroid plexus epithelium. Another transcript with product likely to be involved in the extravasations of peripheral cells, integrin α 6 (*Itga6*) was also expressed at a higher level at the blood-brain barrier and was enriched in the adult when compared to postnatal mice (Daneman et al., 2010). In the choroid plexus *Itga6* transcript, though with lower expression than the cerebral vasculature, was enriched in the embryonic mouse over 7-fold (Liddelow et al., 2013) highlighting the potential developmental difference in the role of the choroid plexus and blood vessels in the contribution to immune surveillance of the brain and the response to inflammation.

The level of peripherally-derived, blood-borne cytokines entering the brain is low, however, it is comparable to other water-soluble molecules that are known to cross the brain barriers to a degree sufficient to affect brain function (e.g., morphine, Banks et al., 1995). There are a large number of transport systems for common inflammatory mediators that are present on both the blood-brain and blood-CSF barriers. IL-1, a pro-inflammatory cytokine, is able to exert a range of effects on the brain, including mediating key host defenses in response to many chronic CNS diseases. The functional family of IL-1 contains the agonists (IL-1α and IL-1β ), the receptors (IL-1RT1 and IL-1RT2) and a naturally occurring antagonist molecule (IL-1RN). At the blood-brain barrier, endothelial cells contain measurable levels of IL-1β and IL-1RT2 (the receptor with a higher affinity for IL-1β ) while levels of transcript for IL-1α and the type 1 receptor (IL-1RT1) fall below levels of detection (Daneman et al., 2010). Levels of transcript do not appear to change through development, at least in the mouse. In contrast choroid plexus epithelial cell expression of IL-1 members shows the predominant receptor transcript that is detected is IL-1RT1, with over a 10-fold increase in expression between the embryo and the adult Liddelow et al., (2012).

Similar to IL-1, IL-6 signals through a cell-surface type I cytokine receptor complex. It is made up of the ligand-binding IL-6RA segment (*Il6r*) and the signal-transduction IL-6RB component (*Il6st*). It should be noted that IL-6RB is also a common signal-transducer for other cytokines (e.g., LIF, CNTF, IL-11, among others). *Il6* ligand transcript is low in endothelial cells, however, *Il6r* and *Il6st* are high from very early in development and do not change into adulthood. A similar lack of developmental expression changes was seen in the choroid plexus with low *Il6* and *Il6st* expression in both embryonic and adult mice Liddelow et al., (2012), however, no expression for *Il6r* was detected in this study. A more recent RNA sequencing study by these authors, however, reports expression of *Il6r* in choroid plexus epithelium (Liddelow et al., 2013), highlighting the importance of validation of microarray genechip experiments to ensure no false positive or negative results.

The levels of transcript for TNFα by barrier cells (both cerebral endothelium and plexus epithelium) are extremely low, suggesting the majority of TNFα in the CNS is provided by local production from other cells types (e.g., microglia—though it is likely they only produce measurable levels of TNFα following injury), or by transport from the periphery. Having said this, following induction of a peripheral inflammatory response, levels of TNFα (as well as IL-1β ) transcript in choroid plexus epithelial cells and meningeal endothelium increased (Quan et al., 1999). Knock-out animal models for TNFα receptors *Tnfrsf1a* (TNFR1/p55 receptor) and *Tnfrsf1b* (TNFR2/p75 receptor) have shown a reduction of the ligand penetrating the blood-brain barrier into the spinal cord, but not into the brain of single knock-out animals (Pan and Kastin, 2002). Double knock-out animals of both *Tnfrsf1a* and *Tnfrsf1b* showed a complete abolition of TNFα penetration—suggesting that both receptors are necessary for transporting the ligand into the CNS (Pan and Kastin, 2002). Genechip data from the blood-brain barrier (Daneman et al., 2010) show a high expression of both *Tnfrsf1a* and *Tnfrsf1b*, as well as several other TNFα receptor family members (*Tnfrs11a*, *12a, 19*, and 21) with no change in expression between early postnatal and adult mice. At the blood-CSF barrier, plexus epithelial expression of TNFα receptor family transcripts is low, however, there is embryonic enrichment of *Tnfsf1b* and *Tnfrsf21*.

It therefore appears that while the barrier systems may not produce a vast array of cytokines under resting conditions, both in development and adulthood, they express many receptors for inflammatory mediators and signal amplifiers, indicating the importance of an early vascular response to inflammatory signaling. Barrier cells also appear able to rapidly up-regulate the expression, and likely release, of some cytokines following an inflammatory insult in as little as a few short hours. The capacity of the barrier cells to respond to inflammatory signaling may be an important confounding factor in the developmental response of the brain to inflammation. While it is beyond the scope of this review, it is important to note that the systemic immune response is also changing over this time, and may contribute to the differences observed in the CNS response to inflammation/injury during development.

## Developmental inflammatory response

It is now clear that the vasculature in the developing brain is primed to respond to inflammatory stimuli. Despite this, little work has been done to investigate the blood-brain barrier response to inflammation throughout CNS development. This is presumably partly due to the historical misconception that the blood-brain barrier is functionally immature in the developing brain. However, it has been well-established (as described above) that the structural and functional mechanism that contribute to the blood-brain barrier are present from very early in embryogenesis. Work from the last 10 years also suggests that the response of the blood-brain barrier to inflammation is selective and specific depending on the age at the time of insult and the location of the inflammatory signals (discussed below).

Work from our laboratories has shown that systemic inflammation causes a specific increase in the permeability of the blood-brain barrier in vessels in the periventricular white matter tract in neonatal rats (Stolp et al., 2005a). The reasons for the increased permeability in these blood vessels is not yet clear, though numerous explanations have been presented, including a developmental delay in the maturity of these vessels or a specific susceptibility to increased vascular flow. Regarding potential immaturity of cerebral vessels, work from Virgintino et al. (2004) and Anstrom et al. (2007) have clearly shown variation in the complexity of tight junction proteins in the microvasculature of the human brain with different developmental ages and brain regions. While the complexity of the tight junctions has been much discussed in the context of brain development, it is not yet clear how well this correlates with barrier permeability (Møllgård et al., 1979). It is suggested that a delayed maturation of the vessel structure in the germinal matrix and periventricular white matter may lead to increased susceptibility of these brain regions to damage during premature birth, hypoxia, or inflammatory insults (Anstrom et al., 2007). This is an appealing hypothesis, which recognizes a maturation process that may be sufficient, for example in the controlled intrauterine environment, for normal function but which could be easily damaged by changes in blood pressure or some other environmental challenge. It has been established, however, that changes in cerebral blood flow are in themselves insufficient to account for damage in these brain regions following hypoxia-ischemia (Mcclure et al., 2008). Additionally, the age specific increase in blood-brain barrier permeability reported by Stolp et al. (2005a) is not easily explained if the complexity of the tight junction structure is the only contributing factor in the vascular response to insult. When a marsupial species was used to repeat experiments studying the age-specific response to inflammation, so that a longer developmental period could be assessed in a postnatal systemic inflammation paradigm, it was determined that the increased permeability of the periventricular white matter vessels was limited to a specific stage of development, rather than a general response of the developing brain (Stolp et al., 2005a). There are two potential explanations for this: the first that the inflammatory response at the earliest times is not sufficiently developed to stimulate the signaling pathway responsible for the increased permeability; or secondly, that there is a specific combination of factors that occur at the equivalent of the first post-natal week in the rat which combine to produce the susceptibility of the barrier in these specific vessels. There is certainly a substantial increase in the number of activated and migrating microglia and astrocytes in the white matter at this stage of development (Stolp et al., 2009; Verney et al., 2010, 2012), which may contribute to the central inflammatory response and increase the sensitivity of the nearby vessels to the inflammatory signals. The transcriptome of astrocytes activated following peripheral LPS inflammation in adults show a marked increase in the expression of many receptors to cytokines such as TNFα and TGFβ, however, there is not the same increase in the expression of the ligands themselves (Zamanian et al., 2012). There is, however, a relatively high expression of the lipocalin 2 receptor, *Slc22a17*, which is not present on choroid plexus epithelium (Liddelow et al., 2013), but is on cerebral endothelium (Daneman et al., 2010) in close association with astrocytic endfeet. Lipocalin 2 is involved in the innate immune response by sequestrating iron, in turn limiting bacterial growth (Yang et al., 2002), and has recently been shown to be the highest enriched transcript in reactive astrocytes (Zamanian et al., 2012), suggesting an astrocytic role in the innate immune system and the acute phase response to infection in the CNS, and therefore a potential for an atypical cerebral inflammatory response when astrocytes are apparently activated by migration during development.

Interestingly, different developmental barrier susceptibility has been identified in response to directly induced intracerebral inflammation. Injection of IL-1β into the striatum of postnatal day 2 (P2), P21, and adult rats produced a substantial difference in the inflammatory response (Anthony et al., 1997). A small increase in neutrophil accumulation was observed at P2 and in adult animals and a small increase in permeability of vessels to horseradish peroxidase within the injection site, as well as in the meningeal vessels. However, in P21 animals there was a significant increase in permeability of all the vessels in the injected hemisphere associated with a substantial increase in neutrophil extravasation into the brain. Subsequent experiments showed that the changes in permeability were neutrophil dependent, as neutrophil depletion by x-irradiation of the bone marrow prevented this response (Anthony et al., 1997). A neutrophil specific alteration in blood-brain barrier permeability has also been described in a model of stroke (Fernandez-Lopez et al., 2012). However, in this case the early postnatal brain appeared to be protected against altered blood-brain barrier permeability and neutrophil infiltration, compared to the adult. While various small changes in vascular structure (e.g., high basal levels of basement membrane proteins) and activation processes (variable adhesion molecule expression following stroke) were recognized, Fernandez-Lopez and colleagues (2012) determined that the reduced response in neonates was not due to a lack of capacity for neutrophil migration in early development, but instead may be due to altered ratios of chemoattractant molecules between the systemic and central systems. This highlights important differences between models of developmental brain injuries and the etiological mechanisms involved. There is a clear need for further research in this area to tease apart specific signaling systems. Particularly given the completely different response to that seen in the neonatal rat following systemic inflammation, where no neutrophil infiltration has been reported in relation to an age and location specific change in barrier permeability (Stolp et al., 2005a).

The observed developmental differences in the CNS response to inflammation are likely to reflect a combination of many aspects of brain development as well as maturation of the systemic inflammatory response. Specific studies are still lacking on the interactions between these two systems in development, as has been done in adult neuroinflammatory disease (see Anthony et al., 2011).

## Consequences for disease and ageing

The consequence of the inflammatory signaling process and the potential association of changes in blood-brain barrier permeability may be widespread in the developing brain. The specific changes within the developing brain appear to vary depending on the timing of insult and reflect a mixture of the developmental stage of the CNS, as well as the specialities of the immune signaling response of the barrier systems at the time of insult.

**Figure 3:**
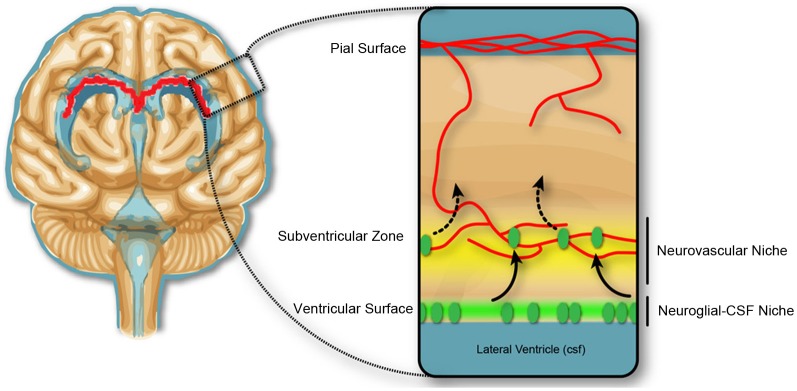
**Neurogenic niches in the developing brain.** Dividing cells in the subventricular zone are closely associated with blood vessels, and the concept of a neurovascular niche (yellow), which reflects a zone of influence of vascular factors on neural progenitor cells, suggested in the adult and developmental subventricular zone. In early development, the dividing cells in the ventricular zone are not closely associated with the blood vasculature, but may be affected by trophic factors produced in the CSF, and therefore exist in a neuroglial-CSF niche. Cells from the ventricular zone migrate toward the neurovascular niche, before differentiating and migrating to outer cortical layers of the brain.

In the second half of gestation in the rodent, equivalent to the 1st—2nd trimester in humans (Clancy et al., 2001), there is no evidence of blood-brain barrier disruption associated with experimentally induced inflammation. However, there is substantial evidence for changes to the developing brain, which reflect changes in immune signaling. There is a reported decrease in (VZ) proliferation, but not the subventricular zone (SVZ), in response to low dose LPS-induced inflammation in mice at E13.5 of gestation (Stolp et al., 2011). The change in proliferation in the VZ but not the SVZ implies a variable contribution of the vasculature and the CSF for central immune signaling following induction of the systemic maternal inflammatory response (discussed further below), and indicate that the progenitor cells in the VZ and SVZ are in different environmental niches (Figure 3). Additional studies confirm the sensitivity of the VZ cell population to immune signaling, indicating presence of receptors to specific cytokines (e.g., IL-1β) or pathogen associated molecules (including TLR2 and 3) and stimulation of these receptors decrease neurogenesis and may alter cellular differentiation (Lathia et al., 2008; Okun et al., 2010; Crampton et al., 2012).

The change in permeability of the ventricular surface in concert with the decreased proliferation in the VZ (Stolp et al., 2011) but an absence of altered permeability at the blood-brain barrier supports the idea of a CSF-brain specific signaling mechanism regulating the proliferation of cells in the VZ in early development. Recent work by Lehtinen et al. (2011) shows that insulin-like growth factor 1 produced by the choroid plexus in late gestation in the mouse modulates proliferation of the VZ progenitors. Additionally, a selective fourth ventricle OTX-induced choroid plexus deletion, which significantly changes the composition of the CSF, also modifies proliferation in the cortical VZ throughout gestation (Johansson et al., 2013). Cunningham et al. (2013) hypothesize that microglia in the developing brain may be integral to the modulation of proliferation in the progenitor zones of the developing brain, however, their observations are true for both the VZ and the SVZ and may reflect an additional level of control, separate to CSF-specific inflammatory signaling pathways. Substantial changes in the number of F4/80 positive monocytes/microglia within the developing brain were not observed in a study of low-dose maternal immune regulation (Stolp HB, unpublished data). The presence of strap junctions between the neuroependymal cells in early fetal development but not in the adult (Møllgård et al., 1987), suggest a developmentally important role of junctions between the progenitor cells in the VZ. It has been suggested that junctions between these cells are important for regulation of polarity and therefore proliferation in the VZ (Huttner and Brand, 1997). It is possible that these junctions are modified in response to inflammation in a similar manner to that described for adult barrier junctions. Although the presence of strap junctions forming the inner CSF-brain barrier is only present early in development, there is still specific uptake of proteins by these neuroependymal VZ cells (Figure 2)—reiterating the importance of protein-cargo trafficking into the CSF and thence the brain during development (Knott et al., 1997; Liddelow et al., 2012).

A different response is seen slightly later in the process of brain development. In early postnatal rodents [approximately P1–7, equivalent to the 2nd—3rd trimester of human pregnancy, (Clancy et al., 2001)] increased permeability of the blood-brain barrier is observed specifically in the periventricular white matter and associated with damage (Stolp et al., 2005a,b, 2009). It is currently unclear how much the damage in this area of the brain is directly related to increased barrier permeability or other associated phenomenon. Large quantities of plasma proteins in the brain, as occurs with blood-brain barrier breakdown, have been associated with increased cell death (Nordborg et al., 1991; Wagner et al., 2002) and altered neuronal function, potentially leading to epileptic-type activity (Friedman, 2011; Tomkins et al., 2011). It is suggested, however, that changes in blood-brain barrier permeability associated with systemic inflammation in postnatal animals is not enough to account for the white matter damage alone, as it requires increased microglial activation (Stolp et al., 2009). Increased numbers of microglia, particularly with the morphological appearance of activation, have been associated with the peak periods of white matter damage (Verney et al., 2010, 2012; Supramaniam et al., 2013). It is possible that the large number of migrating, activated microglia within the white matter tracts are primed to respond to inflammatory signaling transferred through blood vessels, or to the presence of systemic proteins following blood-brain barrier breakdown, and it is these immune cells that interact with oligodendrocytes in the developing white matter to cause injury.

These two scenarios indicate changes to the brain that are an immediate cause in systemic inflammation and inflammatory signaling into the developing brain. There are likely to be many more examples like this, as numerous immune mediators are important for the regulation of maturation processes in the brain (e.g., CXCR4 and CXCL7 as key migration cues). It is, however, necessary to also consider subtle changes that may alter the response of the maturing/ageing brain to insults later in life. One example of this is a long-term alteration in blood-brain barrier function that occurs following systemic inflammation early in life. In this case, the magnitude/prolonged nature of the inflammatory response is key—and long-term changes in barrier function only occur after prolonged exposure to systemic inflammatory (Stolp et al., 2005b).

Given the contribution of the blood-brain barrier to adult neuroinflammatory diseases (as discussed above), any structural deficits within the barrier junctions that exist as a result of injury in early life may increase the risk of early or delayed onset of neurodegenerative conditions [reviewed by Stolp and Dziegielewska (2009)].

## Summary remarks

1. The brain, both in the adult and in development, is surrounded by a complex array of barrier mechanisms comprised of morphological (tight junctions), biochemical, and physiological (influx and efflux transporters) components that control and determine its internal environment. There is increasing evidence that one important function is an interaction with the immune system. Any structural deficits within the barrier junctions that exist as a result of injury in early life may increase the risk of early onset of neurodegenerative conditions.
2. Evidence suggests that normal immune surveillance, which is likely to occur primarily through the blood-CSF barrier, is facilitated by the specific composition of the junctions between epithelial cells. Junctional rearrangement appears to be an essential element of inflammation-induced cellular recruitment to the brain.
3. Transporters constitutively present at brain barriers can be affected by inflammation, therefore contributing to changes in the CNS environment alone or in association with the changes produced by the activation of immune cells.
4. There is increasing evidence that the brain barriers are able to mount a response to peripheral inflammation and that this vascular inflammatory response may in itself contribute significantly to neuroinflammatory disease.
5. The developmentally controlled CNS response to inflammation is a combination of many aspects of maturation processes of both the brain and the systemic inflammatory response itself. Specific study of the interactions between these two systems in development, as has been done in adult disease, is very important for proper understanding of normal and pathological mechanisms involved.

## Conclusion

The brain barrier systems provide an essential interface between the periphery and the brain, which is intrinsically involved in the communication of inflammatory signals between these two compartments. Though very little is known about the responses of individual cells forming these barriers during inflammation, especially during development and ageing, it is apparent that they respond differentially to disease. We can say with confidence therefore that immunity is an active and fluid component of normal brain-barrier function. What we cannot say with a similar level of confidence, however, is how this function is altered under stress, or how one should approach these alterations from a clinical setting. There is still a substantial amount of work required before specific aspects of changes in the plethora of barrier mechanisms contributing to neuropathological conditions arising during development and in old age can be defined. More attention needs to be paid to changes in cellular-based barrier mechanisms, rather than focus on the integrity of tight junctions, which has been the emphasis of much of the research effort in this field so far.

### Conflict of interest statement

The authors declare that the research was conducted in the absence of any commercial or financial relationships that could be construed as a potential conflict of interest.

## Abbreviations

CSF: cerebrospinal fluid
CNS: central nervous system
EAE: experimental autoimmune encephalitis
E: embryonic
Itga6: integrin a6
JAM: junctional adhesion molecule
IL: interleukin
PGP: P-glycoprotein
PKC: protein kinase C
SVZ: subventricular zone
TNF: tumor necrosis factor
VCAM: vascular cell adhesion molecule
VZ: ventricular zone, ZO, zonular occludin.
